# Protective Effect of Vitamin E against Diabetes-Induced Oxidized LDL and Aorta Cell Wall Proliferation in Rat

**DOI:** 10.6091/ibj.1449.2015

**Published:** 2015-04

**Authors:** Alireza Shirpoor, Leila Norouzi, Samira Nemati, Mohammad Hasan Khadem Ansari

**Affiliations:** 1 *Dept. of Physiology, Faculty of Medicine, Urmia University of Medical Sciences, Urmia, Iran;*; 2 *Dept. of Biochemistry, Faculty of Medicine, Urmia University of Medical Sciences, Urmia, Iran*

**Keywords:** Ox-LDL, Vitamin E, Diabetes, VSMC proliferation

## Abstract

**Background:**

Hyperlipidemia and oxidized-low-density lipoproteins (Ox-LDL) are important independent cardiovascular risk factors that have been shown to stimulate vascular smooth muscle cell (VSMC) proliferation. The purpose of the present study was to investigate the effect of vitamin E on Ox-LDL, lipid profile, C-reactive protein (CRP), and VSMC proliferation of rat aorta.

**Methods:**

Male Wistar rats (n = 32) were divided into four groups namely: sham (SH), control (C), non-treated diabetic, and vitamin E-treated diabetic (VETD) groups. Ox-LDL, lipid profile, CRP and VSMC proliferation of aorta were measured after 42 days.

**Results:**

The results revealed that along with a significant increase in VSMC proliferation, the amount of CRP, Ox-LDL, and lipid profiles in diabetic rats. VSMC proliferation was significantly ameliorated, and elevated CRP, Ox-LDL, and lipid profiles were also restored to those of shams in VETD.

**Conclusions:**

These findings strongly support the idea that diabetes induces Ox-LDL-mediated oxidative stress and VSMC proliferation in aorta of rat and imply that vitamin E has a strong protective effect as an antioxidant.

## INTRODUCTION

There is accumulating evidence that suggests the cardiovascular disease (CVD) is the most common serious complication of diabetes mellitus. Duration of diabetes and hyperglycemia, as well as hypertension, dyslipidemia, insulin resistance, gender, and coagulation abnormalities are a number of factors related to an increased risk for CVD [1]. The mechanisms of vascular injuries in diabetic patients have been studied since past decades. However, a clear picture of molecular events, which play crucial roles in the pathophysiological mechanisms, has not been elucidated yet. It has been reported that diabetes may partly act via oxidative stress, creation of oxidized–LDL (Ox-LDL), and its induced vascular disease [2, 3]. So far, the atherosclerogenic effect of Ox-LDL on vascular smooth muscle cells (VSMC) has not fully understood. Accumulation of Ox-LDL in the vascular wall is believed to be a crucial leading mechanism of atherosclerosis which induces activation of macrophages and endothelial cells as well as impairment of the physiologic action of nitric oxide and apoptosis [4]. It also triggers regeneration of oxygen reactive spaces [-]. Pro-inflammatory properties of Ox-LDL have also been reported [8]. Recently, research in the role of modified lipoprotein, preliminarily Ox-LDL in CVD, has been grabbed more attention, and further evidence support that Ox-LDL as well as antibodies against epitopes of Ox-LDL are present in both human and rabbit plasma and in atherosclerotic lesions [8, 9]. Initiation of inflamm-atory mediators, such as C-reactive protein (CRP), IL-6, and tumor necrosis factor could be considered as a further role of Ox-LDL in atherosclerosis [9]. Among these markers, CRP has been widely identified as a non-specific but sensitive marker of the acute inflammatory [10]. CRP is not only a significant prognosticator of future cardiovascular events but also it is a direct participant in the pathogenesis of athero-sclerosis [11]. It is well-known that the activities and expressions of multiple factors implicated in athero-genesis, such as stimulating release of endothelin-1 and IL-6 from endothelial cell, increasing the expressions of adhesion molecules and monocyte chemoattractant protein-1 and facilitating the uptake of low-density lipoproteins (LDL) by macrophage modulates via CRP [10, 12, 13]. Additionally, CRP stimulates migration and proliferation of VSMC [12]. Recent studies have been also proved that coronary artery smooth muscle cells of human and rat produce CRP [14, 15]. Direct participation of the locally produced CRP in atherogenesis and development of cardiovascular complication have been also reported [16]. It has been demonstrated that the earliest event in development of the atherosclerotic lesion in the presence of elevated lipid levels is the transport into and retention of LDL in the artery wall [17]. Consequently, LDL undergoes oxidation and is taken up by macrophages with the subsequent formation of foam cells [17]. The presence of smooth muscle cells, abundance of intercellular matrix, and greater accumulation of macrophages and lipoprotein distinguish progression-prone from progression-resistant lesions [17]. Depend on the concentration and level of LDL oxidation, Ox-LDL has been shown to affect VSMC growth by inducing proliferation or apoptosis [18]. An oxidative mechanism has been also reported that stimulates Ox-LDL growth via the release of fibroblast growth factor-f, potentiation of the mitogenic effect of angiotension II, and induction of an increase in the expression of some cell cycle regulatory proteins [19].

Hence, the current study was sought to elucidate the effects of elevated levels of Ox-LDL and CRP on VSMC proliferation as an indicator of cardiovascular dysfunction in diabetic rat aorta. The effect of vitamin E on attenuation of Ox-LDL-induced VSMC prolifer-ation and CRP mediate inflammatory response was also investigated.

## MATERIALS AND METHODS

All procedures were handled in accordance with the Principles of Laboratory Animal Care (NIH publication no. 85-23, revised 1985) as well as the specific rules provided by the Animal Care and Use Committee of Urmia Medical University and National Medical and Health Service. Male Wistar rats (n = 32, six months old) with initial body mass of approximately 200 ± 20 g were included in this study. Rats were assigned into four groups of eight animals each: sham (SH), control (C), non-treated diabetic (NTD), and vitamin E-treated diabetics (VETD). Diabetes was induced in 16 rats by a single i.p. injection of streptozocin (STZ) (60 mg/kg) (Sigma Aldrich, USA) buffered in cold sodium citrate (pH 4.5). Control rats received an equivalent amount of the buffer. Forty eight hours after the STZ injection, hyperglycemia was determined by measuring the content of tail vein blood glucose using the glucose oxidase-based biosystem kit (Biosystems, Barcelona, Spain). Rats with blood glucose higher than 300 mg/dl were considered as diabetic. According to a preliminary study, rats in the VETD group were gavaged 300 mg of vitamin E (Merck GmbH, Germany) as a non-toxic dose besides the regular diet daily [20]. The control and NTD groups were treated with vehicle only (top water). For elimination of any effect of the intragastrical gavage on the measured parameters, some numbers of the rats without any intervention were assigned as sham. Foods were supplied *ad libitum* to all groups throughout the experiments. After six weeks, all rats were anesthetized i.p. by ethyl carbamate (urethane) (1 g/kg), and depth of anesthesia was assessed by pinching a hind paw. 

***Blood samples. ***Blood samples were collected directly from carotid artery, mixed with EDTA as an anticoagulant, and centrifuged at 1,000 ×g for 10 minutes within 30 minutes of collection. The plasma samples were aliquoted and stored at -80°C without repeated freeze-thaw cycles. Whole blood (1 cc) was collected for hemoglobin A1c (HbA1c) measuring.

***Biochemical assays:***

***C-reactive protein. ***Serum level of CRP was measured by nephelometric methods using Minineph TM according to the protocol provided by the manufacturer (ZK044.L.R, Binding Site Ltd., Birmingham, UK). The approximate measuring range was 3.51-12 mg/L at a sample dilution of 1/40. The sensitivity limit was also 0.44 mg/L when using a 1.5-sample dilution.

***Ox-LDL. ***The plasma level of Ox-LDL was measured using a capture ELISA (also known as a sandwich ELISA) kit, in which the wells of the microtiter plates were coated with the capture antibody (mAb-4E6, Mercodia, Sweden). Blood samples were collected in tubes containing anticoagulant, and the plasma fractions were separated. Samples were stored at -80°C until measurement without repeated freezing and thawing. Diluted plasma samples (1:6561) were used for ELISA measurements, and optical density of the wells was read at 450 nm, and results were calculated.

***Lipid Profile. ***Serum triglyceride and total cholesterol were measured by colorimetric and enzymatic methods. In addition, serum LDL-C and HDL-C were assayed by the direct method using Biosystem kits (Biosystem, Barcellona, Spain). Apoptosis A (apoA) and apoB were measured by nephelometric method using Mono Binding kit (Binding Site, UK) according to kit insert. 

***Hemoglobin A1c. ***HbA1C or glycosylated hemoglobin was analyzed by HPLC using automated D-10 BioRad hemoglobin analyzers (Germany).

***Proliferating cell nuclear antigen (PCNA) staining (immunohistochemistry).*** The thoracic cavity and abdomen of animals were opened, and aorta was dissected from the root to the abdominal descending part. Tissues were fixed in buffered formalin and embedded in paraffin after standard dehydration steps. Tissue sections (4-µm thick) from formalin-fixed paraffin-embedded aorta were deparaffinized by immersing in xylene, rehydrated by gradual ethanol passage, and washed in Tris buffer. Monoclonal rat anti-PCNA antibody (Dako Denmark A/S, Denmark) was used to stain the slides after appropriate Ag retrieval step, and optimal results were achieved by the EnVision™ visualization system (Dako Denmark A/S, Denmark). Hematoxylin was used as counterstain, and appropriate negative controls were included in assessment, and all slides were inspected by two expert pathologists independently. PCNA-positive indices were considered as indicators of proliferation of muscle cells. Scoring was performed in the following fashion: a number of 100 cells were scored from each tissue section for assessing the percentage of PCNA-positive indices. The criteria for quality scoring of PCNA-positive indices were as follows: normal, PCNA-positive indices less than 5%; mild, PCNA-positive indices present in less than 25% of muscle cells; mild to moderate, PCNA-positive indices present in 25% to 50% of muscle cells; moderate to severe, PCNA-positive indices present in 50% to 75% of muscle cells and severe, PCNA-positive indices present in 75% to 100% of muscle cells. The sections were examined under a light microscope, and photomicrographs were taken.

***Statistical analysis. ***Statistical significance between groups was assessed by one-way ANOVA, followed by Tukey's post-hoc test. In each test, the data were expressed as the mean ± S.E.M., and *P* < 0.05 is accepted as statistically significant.

## RESULTS

Table 1 summarizes the effect of treatment of diabetic rats with vitamin E on several parameters. As shown in Table 1, body gain was significantly lower in the NTD rats on days 15, 30, and 42 after the induction of diabetes compared to the control and sham groups (*P* < 0.001). However, there was no significant difference among the VETD rats compared to the control and sham rats (*P* < 0.6). 

HbA1c was significantly elevated in the NTD (*P *< 0.001), which shows persisted hyperglycemic status, but its level was decreased after treatment with vitamin E to the level of the control and sham groups.

The level of plasma cholesterol showed a significant increase in the NTD group at the end of experiment compared with the sham and control groups (Table 1). There were no significant differences between the control and sham groups as well as among the VETD group and the control and sham groups.

The values of triglycerides were increased significantly in the NTD group compared to the control and sham groups (*P* < 0.001), but triglyceride values were restored in the VETD group as compared to the control and sham groups (*P* < 0.02).

**Table 1:** Result of body gain, HbA1c and lipid profile of study groups

**Parameters**	**Sham**	**Control**	**NTD**	**VETD**
Body gain_15_ (g)	2.00 ± 0.75	2.80 ± 0.68	-18.25 ± 4.38*	-2.62 ± 2.70*†
Body gain_30_ (g)	6.50 ± 0.82	6.50 ± 0.94	-17.60 ± 4.20*	9.00 ± 4.60†
Body gain_42_ (g)	11.50 ± 1.47	11.37 ± 1.34	-24.10 ± 5.90*	6.25 ± 6.22†
HbA_1_c (%)	9.38 ± 0.73	9.50 ± 0.55	29.80 ± 1.00	9.95 ± 0.85†
Cholesterol (mg/dl)	48.40 ± 2.60	48.80 ± 1.90	70.58 ± 3.50*	54.25 ± 2.70†
Triglyceride (mg/dl)	30.85 ± 1.00	31.14 ± 0.96	53.28 ± 3.80*	35.00 ± 3.10†
HDL (mg/dl)	31.57 ± 1.60	30.70 ± 2.27	42.2.10 ± 2.10*	36.00 ± 2.40
LDL (mg/dl)	27.70 ± 1.40	25.70 ± 2.30	36.00 ± 3.50*	29.30 ± 2.20†
Apo-A (mg/l)	138.50 ± 4.50	13.00 ± 8.20	93.80 ± 1.24*	123.00 ± 2.07†
Apo-B/Apo-A	0.86 ± 0.04	0.79 ± 0.040	1.70 ± 0.06*	0.94 ± 0.30†
Blood glucose 48 h after STZ injection	147.00 ± 2.60	148.00 ± 3.20	462.00 ± 24.70*	44.00 ± 16.80*
Blood glucose at the end of experiment	146.80 ± 2.90	150.00 ± 2.70	510.00 ± 12.90*	283.00 ± 16.70*†

Values are expressed as mean ± SEM.

* Significant difference compared to control and sham.

† Significant difference compare to the NTD (non-treated diabetic). VETD, vitamin E-treated diabetics; Apo, apoptosis; STZ, streptozocin

**Fig. 1:**
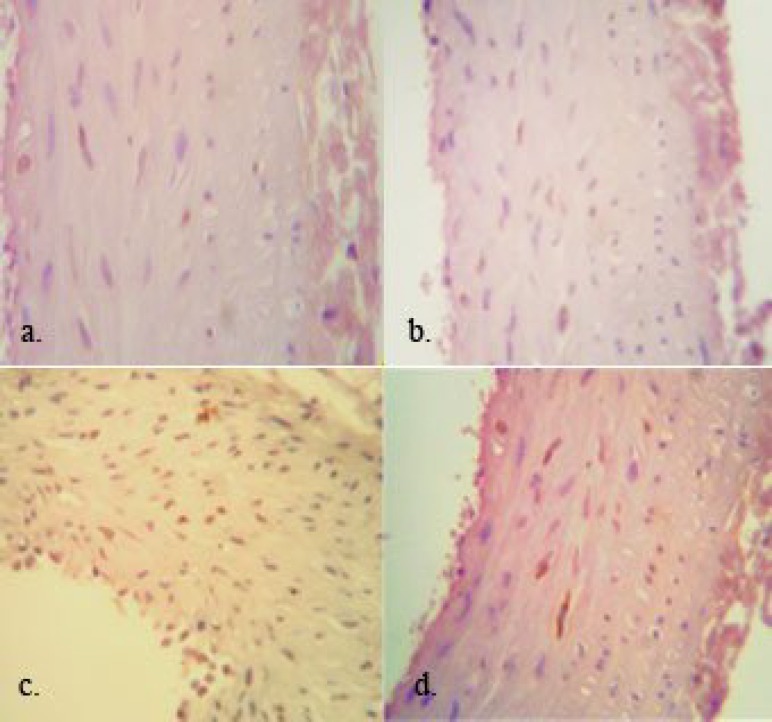
Photomicrographs showing PCNA-positive cells expression in aorta of different groups (magnification ×400). A, sham; b, control; c, NTD (non-treated diabetic); d, VETD (vitamin E-treated diabetics)

The plasma LDL level was increased in the NTD group compared to the control and sham groups (*P *< 0.03), but LDL level diminished to the level of the control and shams in the VETD group (*P* < 0.9). After six weeks, the levels of HDL were increased significantly in the NTD and VETD groups compared to the control and sham groups (*P* < 0.05), but no significant differences were observed between the VETD and NTD groups.

After 48 hours of STZ administration, blood sugar showed a significantly increase in the NTD- and VETD-treated groups compared to the control and sham groups (*P* < 0.05). After 42 days of treatment, blood sugar level in the VETD group was significantly decreased compared to the NTD group (*P* < 0.05), but this level was significantly higher than in control and sham groups (*P* < 0.05).

The plasma apoA levels in diabetic group were significantly lower than those in the sham and control groups (*P* < 0.001), but there was no significant differences between the VETD group and the control and sham groups (*P* < 0.9). Plasma apoB contents were significantly higher in the NTD rats compared to the control and sham groups (*P* < 0.001). In addition, no differences in plasma apoB levels were found among the VETD, control and sham groups (*P* < 0.7).

***Aorta smooth muscle cell proliferation. ***Typical light micrographs of aorta artery from the sham, control, NTD, and VETD animals at the end of experiment are shown in Figure 1. Very low PCNA-positive indices indicated very low proliferation in aorta smooth muscle cells in the sham and control animals (3 ± 1 and 2 ± 1%, respectively). In diabetic animal, 35 ± 4% of the cells showed PCNA-positive indices and it was more significant higher than the control and sham (*P* < 0.005). The number of PCNA positive cells was significantly decreased in the VETD rats as compared to the NTD rats (8 ± 2%, *P* < 0.005). However, the number of PCNA-positive cells in VETD group was significantly higher than the sham and control rats (*P* < 0.05).

**Fig. 2:**
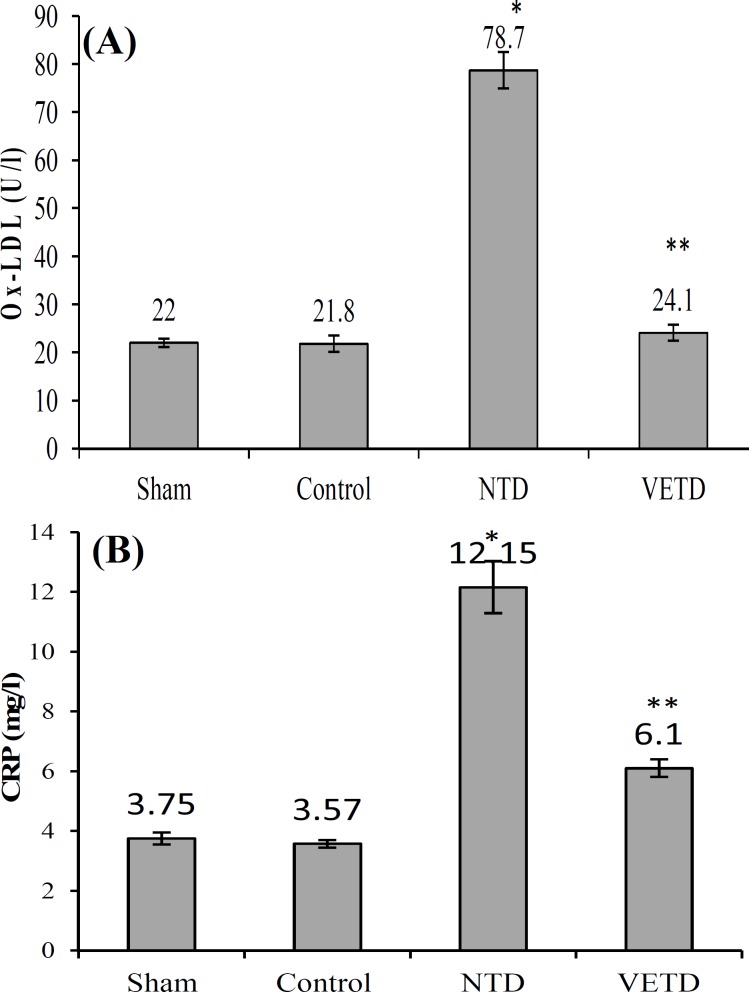
Levels of Ox-LDL (oxidized-low-density lipoproteins) and CRP (C-reactive protein) contents in plasma of different groups. Ox-LDL (A) and CRP (B) contents of plasma in the non-treated diabetic rats were significantly higher. A significant decrease of Ox-LDL (A) and CRP (B) contents was observed after treatment with vitamin E. Values are expressed as mean ± SEM. *Significant difference compared to control and sham; **Significant difference compared to the NTD

***Ox-LDL and CRP. ***Plasma Ox-LDL levels did not differ between the sham and control groups (Fig. 2A). As shown in Figure 2A, the plasma level of Ox-LDL was significantly higher in the NTD group compared with the sham and control groups (*P* < 0.005). There is also no significant differences among the VETD, sham, and control groups (*P* < 0.9). CRP protein levels showed a significant increase in the NTD compared to the sham and control rats (*P* < 0.005), but they were restored in the VETD rats as compared to the control group (Fig. 2B).

## DISCUSSION

Numerous studies have reported the associations between the elevated levels of Ox-LDL and CRP and increased likelihood of cardiovascular events, including hypertension and endothelial dysfunction [3, 21]. In spite of high cardiovascular morbidity among diabetic patients [22], the impact of the elevated level of these biomarkers on vascular cell wall proliferation has not been substantially investigated. The current study shows that diabetic rats suffer elevated levels of both Ox-LDL and CRP in addition to increased levels of cholesterol, LDL, triglyceride, HDL, apoB and decreased level of apoA. In addition, our study demonstrates that there is a significant association between circulating levels of both Ox-LDL and CRP with aorta smooth muscle cell proliferation. The effect of Ox-LDL and CRP on the risk of cardiovascular disease has been reported [3, 23]. Diabetes-induced vascular disorders can be mediated by perturbation of pathways of these biomarkers. Ox-LDL regulates the expression of some genes such as redox-sensitive transcription factors activator-1, nuclear factor-κB, mitogen-activated protein kinase, the vascular gene expression of intercellular adhesion molecule-1 and vascular cell adhesion molecule-1, which are connected with pathogenesis of atherosclerosis [24]. 

The oxidized fatty acids contained in Ox-LDL, 1З-hydroperoxyoctadecadienoic acid and 13-(Ѕ)-hydroxy octadienoic acid, are ligands for peroxisome proliferator-activated receptor γ, consequently its expression could be activated by Ox-LDL [25]. In addition, Ox-LDL stimulates the generation of oxygen reactive spaces [6]. Although the expression of the above mentioned genes was not investigated in the current study, we showed that the well-known antioxidant vitamin E was able to prevent diabetes-induced Ox-LDL elevation and it was related to vascular cell proliferation, indicating that a redox-sensitive signal pathway plays a critical role in complications of diabetes. We observed that the level of cholesterol, LDL, triglyceride, and apoB were increased significantly with the increase in the concentration of Ox-LDL in the serum of rats. LDL particles, native or oxidized, have mitogenic effect on smooth muscle cells [26]. In addition, Ox-LDL is able to induce smooth muscle cell proliferation via gener-ating phospholipase D related second-messengers, which modulate mitogenesis [27]. 

On the other hand, Ox-LDL particles have been also shown that to increase apoptosis of vascular endothelial cells, thereby influencing the pathogenesis of atherosclerosis [28]. Recently, Lamharzi and colleagues [29] have reported that hyperglycemia is not sufficient for stimulating macrophage proliferation in atherosclerotic lesions by itself. Therefore, they proposed that hyperglycemia with concomitant hyperlipidemia is necessary for development of atherosclerosis in diabetic patients. Furthermore, exacerbation of hyperglycemia-related changes in the vasculature and accumulation of Ox-LDL in the plasma of diabetic and cardiovascular disease patients was observed in sever hyperlipidemia [30]. Actually, it is well-known that higher total serum cholesterol and LDL are associated with increased cardiovascular disease risk and mortality [31], and these simple markers are widely accepted as markers for disease risk [32]. 

In the current study, an elevated level of lipid profiles with high plasma Ox-LDL level was observed in diabetic rats with vascular cell proliferation. Our findings prove that impairment of vascular function in diabetes mellitus is directly associated with mutual impact of hyperglycemia and hyperlipidemia, which generates non-enzymatic glycation and oxidation of proteins and lipids like as Ox-LDL. The apoA and apoB amounts in the NTD rats were significantly different from those in the control and sham groups. ApoA levels were significantly decreased and apoB levels were significantly increased in the NTD group when compared with those in the control and sham groups. apoA and B serve as proteins for the very LDL spectrum (LDL, VLDL, and lipoprotein) as well as HDL and determine the metabolic fate of these lipoproteins [33]. Lipoproteins containing apoB carry lipids from the liver and intestine to other tissues that consume the lipids, whereas those that contain apoA mediate reverse cholesterol transport and carry excess cholesterol from peripheral tissues to the liver [33]. As discussed by Walldius and Jungner [34], there are advantages in measuring apoB and apoA [34]. The concentrations of apoA and apo-B indicate the number of their respective lipoprotein particles and the opposite aspects of risks [34]. A high apoB/apoA ratio indicates that atherogenic lipoprotein particles are high and can possibly be deposited in the arterial wall [34]. 

This study showed that a significant increase in apoB level and in the apoB/apoA ratio along with vascular endothelial proliferation in the diabetic group may help the deposition of lipids in the arteries and the consequent arterial complications. Another interesting result of the current study is the fact that higher CRP levels were found in diabetic rats, though the values are considered as inflammatory reaction occurrence in diabetes mellitus. CRP can be used to predict cardiovascular events in patients with stable angina pectoris and in apparently healthy people, through revealing local inflammation in atherosclerosis [35]. CRP, as an inflammatory factor, is used as sensitive but non-specific marker of the acute inflammatory response. It is well-known that CRP, which promotes the activities and expressions of multiple factors, is implicated in atherogenesis (e.g. IL-6 and adhesion molecules) and also facilitate the LDL uptake by macrophage [14, 17, 20]. In addition, it stimulates migration and prolifer-ation of smooth muscle cells in vessels. Another finding of this investigation was that vitamin E not only attenuates diabetes-induced Ox-LDL and CRP elevation but also alleviates aorta VSMC proliferation. The potential preventive effect of vitamin E on oxidative stress-induced disorders in diabetic animals as well as ethanol-mediated oxidative stress complic-ations had been pointed in our earlier public-ations and others [36]. Thus, the current results support the antioxidant role that has been attributed to vitamin E by previous studies. This effect of vitamin E may be related to its antioxidant property, which consequently holds back accumulation of free radicals or toxic materials and induction of proliferation. In addition, an anti-inflammatory effect of vitamin E is well-documented by previous study [20]. In agreement to our study, Devaraj *et al*. [37] study showed that alpha-tocopherol supplementation decreased serum CRP level in normal volunteers and type 2 diabetic patients. Vitamin E supplementation also attenuates the elevation in pro-inflammatory cytokines in humans [37], while minimizing muscle atrophy following limb immobilization and hind limb suspension in experimental animal studies [38].

In conclusion, this study shows that consumption of vitamin E is able to attenuate elevated plasma lipid profile and reduce plasma levels of CRP and Ox-LDL in diabetic rats. In addition, the result of immune-histochemical staining also proved that the expressions of PCNA in the aorta smooth muscle cells in the VETD rats were significantly lower than the NTD animals. Therefore, a combination of oxidative stress and inflammation may contribute to VSMC prolifer-ation in diabetes, perhaps by pathway that involves the promotion of LDL oxidation by glucose.
